# P-type transport ATPases in *Leishmania* and *Trypanosoma*

**DOI:** 10.1051/parasite/2019069

**Published:** 2019-11-29

**Authors:** John C. Meade

**Affiliations:** 1 Department of Microbiology and Immunology, School of Medicine, University of Mississippi Medical Center 2500 North State Street Jackson MS 39216 USA

**Keywords:** P-type ATPase, *Leishmania*, *Trypanosoma*, Trypanosomatid

## Abstract

P-type ATPases are critical to the maintenance and regulation of cellular ion homeostasis and membrane lipid asymmetry due to their ability to move ions and phospholipids against a concentration gradient by utilizing the energy of ATP hydrolysis. P-type ATPases are particularly relevant in human pathogenic trypanosomatids which are exposed to abrupt and dramatic changes in their external environment during their life cycles. This review describes the complete inventory of ion-motive, P-type ATPase genes in the human pathogenic Trypanosomatidae; eight *Leishmania* species (*L*. *aethiopica*, *L*. *braziliensis*, *L*. *donovani*, *L*. *infantum*, *L*. *major*, *L*. *mexicana*, *L*. *panamensis*, *L*. *tropica*), *Trypanosoma cruzi* and three *Trypanosoma brucei* subspecies (*Trypanosoma brucei brucei* TREU927, *Trypanosoma brucei* Lister strain 427, *Trypanosoma brucei gambiense* DAL972). The P-type ATPase complement in these trypanosomatids includes the P_1B_ (metal pumps), P_2A_ (SERCA, sarcoplasmic-endoplasmic reticulum calcium ATPases), P_2B_ (PMCA, plasma membrane calcium ATPases), P_2D_ (Na^+^ pumps), P_3A_ (H^+^ pumps), P_4_ (aminophospholipid translocators), and P_5B_ (no assigned specificity) subfamilies. These subfamilies represent the P-type ATPase transport functions necessary for survival in the Trypanosomatidae as P-type ATPases for each of these seven subfamilies are found in all *Leishmania* and *Trypanosoma* species included in this analysis. These P-type ATPase subfamilies are correlated with current molecular and biochemical knowledge of their function in trypanosomatid growth, adaptation, infectivity, and survival.

## Abstract (fr)

Les ATPases de type P sont essentielles au maintien et à la régulation de l’homéostasie des ions cellulaires et de l’asymétrie des lipides membranaires en raison de leur capacité à déplacer les ions et les phospholipides contre un gradient de concentration en utilisant l’énergie de l’hydrolyse de l’ATP. Les ATPases de type P sont particulièrement utiles chez les trypanosomatidés pathogènes pour l’homme, qui sont exposés à des changements abrupts et dramatiques de leur environnement externe au cours de leur cycle de vie. Cette revue décrit l’inventaire complet des gènes d’ATPase de type P à motif ionique chez les Trypanosomatidae pathogènes pour l’homme ; huit espèces de *Leishmania* (*L*. *aethiopica*, *L*. *braziliensis*, *L*. *donovani*, *L*. *infantum*, *L*. *major*, *L*. *mexicana*, *L*. *panamensis*, *L*. *tropica*), *Trypanosoma cruzi* et trois sous-espèces de *Trypanosoma brucei* (*Trypanosoma brucei brucei* TREU927, *Trypanosoma brucei* Lister souche 427, *Trypanosoma brucei gambiense* DAL972). Le complément ATPase de type P dans ces trypanosomatidés comprend les sous-familles P_1B_ (pompes métalliques), P_2A_ (SERCA, ATPases calciques du réticulum sarcoplasmo-endoplasmique), P_2B_ (PMCA, ATPases calciques de la membrane plasmique), P_2D_ (pompes Na^+^), P_3A_ (pompes H^+^), P_4_ (translocateurs des aminophospholipides) et P_5B_ (sans spécificité attribuée). Ces sous-familles représentent les fonctions de transport des ATPases de type P nécessaires à la survie des trypanosomatidés, car les ATPases de type P de chacune de ces sept sous-familles sont présentes chez toutes les espèces de *Leishmania* et de *Trypanosoma* incluses dans cette analyse. Ces sous-familles d’ATPases de type P sont corrélées aux connaissances moléculaires et biochimiques actuelles sur leur fonction dans la croissance, l’adaptation, l’infectivité et la survie des trypanosomatidés.

## Introduction

Human infection by insect-borne parasites of the family Trypanosomatidae (*Leishmania* and *Trypanosoma*) is a significant public health problem with widespread disease and limited therapeutic options. Leishmaniasis is endemic in 97 countries, putting 350 million people at risk, and there are an estimated 12 million individuals currently infected, with 700,000–1,000,000 new cases and 26,000–65,000 deaths worldwide each year [177, 178]. Clinical manifestations of leishmaniasis include localized skin lesions (cutaneous leishmaniasis), erosion of nasal and oropharyngeal mucosa (mucocutaneous leishmaniasis) or, in visceral leishmaniasis, dissemination throughout the host reticuloendothelial system; as intracellular forms in the spleen, liver and bone marrow. Visceral leishmaniasis (VL) causes 50,000–90,000 new infections annually and the development of clinical disease is generally fatal if untreated. *Leishmania*-HIV co-infection is a serious and growing problem in many areas as HIV dramatically increases the risk of fulminant VL, and VL promotes the clinical progression of HIV [84]. Chagas disease, infection with *Trypanosoma cruzi*, is a major illness in Latin America with 6–7 million infected individuals, 25 million at risk of infection, and an annual death toll of over 10,000 [179]. It is also an emerging public health problem in the United States, Canada, and Europe due to immigration; an estimated 300,000 infected individuals reside in the United States and 67,000 live in Spain [17, 57]. Acute infection from *T. cruzi* trypomastigotes circulating in the bloodstream can be mild to severe with fatalities resulting from myocardial damage. Chronic Chagas disease occurs after trypomastigotes enter cells, particularly myocardial cells, to grow as intracellular amastigotes. Clinical manifestations can appear decades later and include chronic chagasic heart disease (cardiomegaly, dysrhythmias, and cardiomyopathy), due to destruction of cardiac innervation and myocardial cells, and chronic gastrointestinal disease (megaesophagus and megacolon) caused by impaired autonomous neuronal regulation. *Trypanosoma brucei rhodesiense* and *T. b. gambiense* are causative agents of human African trypanosomiasis (HAT) or “sleeping sickness” and if untreated the disease is generally fatal. As recently as 2006, HAT infected 50,000–70,000 people annually, but through the sustained efforts of the World Health Organization and public health officials in affected countries, the annual burden of disease was reduced to less than 3000 cases in 2015 [24, 176]. In HAT infection, circulating trypomastigotes initially cause perivascular leukocytosis and inflammation of the lymph nodes, spleen, vascular epithelium, and endocardium, with death often the result of myocardial damage. The terminal stage of “sleeping sickness” is the result of advanced neurologic involvement as trypomastigotes enter the brain and cerebrospinal fluid (CNS). Infection with *T. b. rhodesiense* is rapidly fatal (weeks) with early CNS involvement and recurrent waves of high parasitemia. *Trypanosoma brucei gambiense* infection produces a chronic disease with low blood parasitemia and late CNS involvement that ends fatally years later. Each of these trypanosomatid infections can be associated with serious medical complications and fatal outcomes, even when treatment is administered. Chemotherapeutic interventions for these diseases are inadequate due to toxic side effects and drug resistance to the current treatment regimens, and there is an urgent need for improved therapeutic alternatives.

These organisms have a complex digenetic life cycle with different morphologic forms in the human host and within the insect vectors of the disease; sandflies for *Leishmania*, tsetse flies for African trypanosomes, and reduviid bugs for *Trypanosoma cruzi* infection. The *Leishmania* life cycle alternates between intracellular amastigote stages in the mammalian host and procyclic promastigotes and non-dividing infectious metacyclic promastigotes in the insect vector. The *Trypanosoma cruzi* life cycle also has intracellular amastigote stages and trypomastigotes present in the mammalian host and epimastigote and non-dividing infectious trypomastigote forms in the insect vector. The *Trypanosoma brucei* life cycle includes procyclic trypomastigotes, epimastigotes, and infectious metacyclic trypomastigotes in the insect host and both dividing (slender) and non-dividing (stumpy) trypomastigote forms in the mammalian host.

Adaptation of the trypanosomatids to these differing environs, and the abrupt transitions that occur, present a challenge for the parasites to adjust to the changing ionic environments and to the structural modifications required for their morphologic changes. The maintenance of intracellular ion homeostasis is critical to growth and survival in all organisms. Proper ionic balance is required for a wide array of cellular processes including regulating osmolarity and cell volume, maintaining pH homeostasis, controlling levels of toxic ions such as heavy metals, providing co-factors for protein function and cellular signaling pathways, and establishing membrane potentials to energize secondary transport systems. To counter the different ionic environments they encounter, cells have evolved a diverse array of proteins to regulate and move ions across both internal and external cellular membranes. These include passive systems such as ion exchangers, ion symporters, and ion channels, as well as active transport systems which require energy for ion movement. The crucial players in this regulation of cellular ion homeostasis are the P-type ATPases which are the only ion transport proteins capable of moving ions against a concentration gradient and thus can facilitate and coordinate the activity of other ion motive mechanisms. This study examines the complete genomes of the human pathogenic Trypanosomatidae in order to identify their complement of P-type ATPases and to correlate current molecular and biochemical knowledge of their function with trypanosomatid adaptation and survival.

## P-type ATPases

P-type ATPases are ubiquitous in nature and have been described in archaebacteria, eubacteria, protozoa, fungi, plants, invertebrates and vertebrates. Proteins of the P-type ATPase family utilize the energy of ATP hydrolysis to transport ions across plasma and organellar membranes and to generate membrane lipid asymmetry [27, 119, 159]. P-type ATPases transport heavy metal ions, K^+^, H^+^, Na^+^, Ca^2+^, and Mg^2+^ ions, and are capable of translocating large phospholipid molecules across membranes. P-type ATPases are typically inhibited by orthovanadate. P-type ATPases are multi-domain membrane proteins with molecular masses ranging from 70 kDa to 150 kDa and share conserved sequence motifs and a common structural organization. All of the P-type ATPases have a similar tertiary structure in their catalytic subunits and a common reaction cycle with two conformational states (E_1_ and E_2_), characterized by the reversible phosphorylation of a conserved aspartate residue, which is part of the phosphorylation site motif DKTGT. The P-type ATPases contain 6–13 hydrophobic transmembrane domains (TMs), typically with cytoplasmic exposure for the amino and carboxyl termini of the proteins, which may contain regulatory elements. P_1A_-ATPases contain 7 TMs, P_1B_-ATPases have 6–8 TMs, P_2_, P_3_ and P_4_-ATPases each have 10 TMs, and P_5-_ATPases have 11–13 TMs [159]. The catalytic core of P-type ATPases consists of six membrane-spanning domains (TM1–TM6) and three hydrophilic, cytosolic functional domains designated as the B-domain or actuator domain, the C-domain, and the J-domain or hinge domain. Nine distinct amino acid motifs, conserved in the P-type ATPase superfamily, have also been identified: PGD, PAD, TGES, PEGL, DKTGTLT, KGAPE, DPPR, MVTGD, and VAVTGDGVNDSPALKKADIGVAM [106]. The B-domain, between TM2 and TM3, contains the first three motifs (PGD, PAD, TGES) and functions to stabilize the transition between the E1 and E2 states. The PEGL motif is found near the end of TM4 and probably contributes to energy transduction. The C-domain contains the DKTGTLT motif which includes the transiently phosphorylated aspartyl residue, the KGAPE motif which participates in ATP binding, the DPPR motif which is involved in phosphate binding and phosphorylation, and the MVTGD motif that is also essential for enzyme phosphorylation. The VAVTGDGVNDSPALKKADIGVAM motif is within the J-domain, which forms a flexible hinge region to allow the conformational changes necessary for ion translocation. Structural analysis indicates that the ion binding sites are located within the intramembrane regions of the pumps. These sites are accessible to the cytoplasm side in the E1 conformation, and accessible to the extracellular side in the E2 conformation [23, 106].

Based on shared sequence homologies, a phylogenetic analysis of P-type ATPases has identified five major evolutionary subfamilies (designated P_1_–P_5_) with P_1_, P_2_, P_3_, and P_5_-ATPases further subdivided into eleven additional classes. Each of these subfamilies and classes is characterized by unique substrate specificity and group-specific sequence motifs [8]. An alternative classification based on the International Union of Biochemistry and Molecular Biology (IUBMB) conventions for transporter classification identifies 9 functionally characterized and 20 functionally uncharacterized families in the P-type ATPase superfamily designated 3.A.3.1–3.A.3.25, 27, 30–32 [142, 159]. This report uses the P_1_–P_5_-ATPase classification of Axelsen and Palmgren [8] as it is much more widely utilized. However, where appropriate, the nomenclature of the IUBMB is also included for clarity, identified as P-type ATPase (3.A.3.) families 1–32 for the transporter classification database (TCDB), where they are maintained (http://www.tcdb.org). The P_1A_-ATPases (3.A.3.7) are bacterial membrane transporters of K^+^ ions. The P_1B_-ATPases (3.A.3.5 and 3.A.3.6) transport heavy metals such as copper, cadmium, lead, and zinc. The P_2_ subfamily transports non-heavy metal cations; P_2A_- and P_2B_-ATPases (3.A.3.2) transport Ca^2+^ ions and are located in internal organelles, particularly the sarcoplasmic and endoplasmic reticulum (SERCA), and in the plasma membrane (PMCA) respectively, P_2C_-ATPases (3.A.3.1) are Na^+^/K^+^- and H^+^/K^+^-ATPases, and P_2D_- and P_2E_-ATPases (3.A.3.9) function as either efflux or influx proteins, respectively in the transport of K^+^ and Na^+^ ions. The P_3_-ATPases translocate H^+^ (P_3A_, 3.A.3.3) and Mg^2+^ (P_3B_, 3.A.3.4) ions. The P_4_-ATPases (3.A.3.8) are phospholipid “flippases” which maintain membrane lipid asymmetry by imparting selectivity and directionality to lipid bilayer movement. The final subfamily, consisting of P_5A_ and P_5B_-ATPases (3.A.3.10–3.A.3.22), does not have an identified substrate specificity or biological function although their involvement in protein maturation, protein secretion, and in anion transport has been proposed.

Although counter-transport of ions has only been documented for the P_2A_ PMCA pumps, the P_2B_-SERCA pumps, and the P_2C_ Na^+^/K^+^-ATPases and gastric H^+^/K^+^-ATPases, functional and structural considerations strongly suggest that counter-transport of ions is mandatory in all P-type ATPases [113, 115]. Transport by the P_2C_ Na^+^/K^+^-ATPases, the P_2B_-SERCA, and the P_3B_ proton pumps is electrogenic in nature, and creates an electrochemical gradient that in the case of P_2C_ and P_3B_ pumps functions to drive secondary transport systems [113, 119]. Ion transport by the remainder of the P-type ATPases appears to be electroneutral. The majority of the P-type ATPases consist of a single catalytic unit and do not require the participation of accessory molecules for activity. However, the P_2C_ Na^+^/K^+^- and H^+^/K^+^-ATPases are heterodimers composed of a catalytic α-subunit and a ~40 kDa, glycosylated, regulatory β-subunit whose co-translational association with the α-subunit is absolutely required for protein maturation, membrane localization, stability and function of P_2C_ ATPases. The P_2C_ Na^+^/K^+^-ATPases can be further modified by a third subunit, the gamma or FXYD subunit, which affects substrate affinity and pump activity [132]. The P_4_-ATPase subfamily of aminophospholipid translocases also requires accessory β-subunit proteins for both correct trafficking to the membrane and lipid translocation activity [120].

## P-type ATPase search criteria

This review examines the genome sequences of *Leishmania aethiopica, L. braziliensis*, *L. donovani, L. infantum*, *L. major, L. mexicana*, *L. panamensis*, *L. tropica*, *Trypanosoma cruzi*, *Trypanosoma brucei rhodesiense*, and *T. b. gambiense* maintained in the TriTrypDB database of the EuPathDB database (http://tritrypdb.org), to identify the complete inventory of P-type ATPases in these organisms [7, 18, 43, 45, 46, 67, 68, 86, 121, 139]. The parasite genomes were searched for proteins containing P-type ATPase amino acid signature motifs for the ATP phosphorylation site DKTGT, the Mg^+^-ATP binding site DGVND, the actuator domain LTGES, and other highly conserved P-type ATPase motifs, DPPR, KGAP and MLTGD. The annotation for predicted protein sequences was also queried for the terms ATPase, P-type ATPase, ion translocation, cation transport, and phospholipid translocation. The P-type ATPase sequences identified were utilized to search for additional homologous genes in these trypanosomatid genomes. As P-type ATPases were found, they were sequentially numbered using the designations of LA, LB, LD, LI, LM, LMX, LP, LT, TB, TBG, and TC for *Leishmania aethiopica, L. braziliensis*, *L. donovani, L. infantum*, *L. major, L. mexicana*, *L. panamensis*, *L. tropica*, *Trypanosoma brucei*, *T. b. gambiense*, and *T. cruzi* ATPases, respectively. The P-type ATPases identified in these searches were subjected to a phylogenetic analysis based on the method of Axelsen and Palmgren [8] which extracts the 265 amino acids contained in eight amino acid signature sequences conserved in all P-type ATPases. These trypanosomatid sequences and those from P-type ATPases representative of other prokaryotes and eukaryotes were used to generate evolutionary trees using the phylogenetic inference program PHYLIP version 3.69 [50, 51]. This analysis coupled with the presence of motifs and motif spacing characteristic of different ATPase subfamilies, allowed assignment of the trypanosomatid sequences to the appropriate P-type ATPase subfamilies. The PubMed, GenBank, and the UniProtKB databases were queried to identify P-type ATPases previously described in the trypanosomatid parasites.

## P-type ATPases in the Trypanosomatidae

A total of 42 complete P-type ATPase protein sequences, listed in Table 1, were identified in the three trypanosomatid genomes initially reported in 2005; 16 in *L. major* (strain Friedlin), 12 in *T. cruzi* (strain CL Brener) and 14 in *T. brucei* TREU927 (strain 927/4 GUTat10.1) [18, 45, 46, 67]. Since that time, genome sequences for seven additional *Leishmania* species and two more *Trypanosoma* species have been reported and are included in this analysis [7, 43, 68, 86, 121, 139]. Available sequence data for other *Leishmania* and *Trypanosoma* species were either from non-human pathogens or were incomplete as judged by the absence of functional genes for some essential P-type ATPase subfamilies and were not analyzed. The *T. cruzi* CL Brener stain used for genome sequencing is a hybrid of two distantly related *T. cruzi* lineages, designated as Esmeraldo-like and Non-Esmeraldo-like, which initially complicated assignment of chromosome specificity [94, 173]. As a consequence, chromosomal alleles for both Esmeraldo-like (s) and Non-Esmeraldo-like (p) strains are included in the genome data for five *T*. *cruzi* ATPases, each represented as nearly identical copies (>97%) on separate contigs (TC1s/TC1p, TC3s/TC3p, TC7s/TC7p, TC11s/TC11p, and TC15s/TC15p), and these duplications are only included once each in the P-type ATPase count for *T. cruzi* strain CL-Brenner. However, the true complement of P-type ATPases in *T. cruzi* is likely 14–15, as partial sequences for several homologues of H^+^-ATPase TC8 were present in different chromosomal locations in the CL-Brener genome sequence and other investigators have reported tandemly linked arrays of three H^+^-ATPase genes in *T. cruzi* Y strain and four H^+^-ATPase genes in the Sylvio X10/7 strain [89, 101]. The differences in the P-type ATPase complement between *L*. *major* and the two *Trypanosoma* species are due to the absence of homologues for calcium motive ATPase LM6 in *T*. *brucei* and *T*. *cruzi* and for proton motive ATPase LM10 in *T*. *brucei*, as well as variable numbers of repeated genes in the additional calcium and proton ATPase loci. The P-type ATPases of the trypanosomatid species were found to be highly syntenic, showing considerable conservation in the surrounding gene order as was true for most of their genome sequence.

**Table 1:** P-type transport ATPases in the originally sequenced genomes of the Trypanosomatidae, 2005.

Protein	Size (aa)	Chromosome	Accession	Protein	Size (aa)	Chromosome	Accession
P1B, Cu^++^, Co^++^ transport				P3A, H^+^ transport			
LM1	1163	33	Q4Q3X8	LM9	974	18	Q4QDN7
TB1	961	11	Q387C6	TB9	912	10	Q388Z3
TC1s/1p	954	39-S, 39-P	Q4DIX9, Q4D866	TC9s	219 (p)	40-S	Q4CP01
				TC9p	484 (p)	40-P	Q4CNY1
P2A, Ca^++^ transport (SERCA)							
LM2	1023	4	Q95Z93	LM10	952	4	O97198
TB2	1011	5	Q57Z55	TB10	Absent		
TC2s	1006	21-S	Q4DIM7	TC10s	898	8-S	Q4DE37
P2B, Ca^++^ transport (PMCA)				P4, aminophospholipid translocation			
LM3	1104	7	Q4QIM8	LM11	1279	30	Q4Q767
TB3	1080	8	D6XMU9	TB11	1196	6	Q584Z5
TC3s/3p	1101, 1103	19-S, 19-P	Q4E3S6, Q4DSJ2	TC11s/11p	1217	32-S, 32-P	Q4DXI9, Q4DT76
LM4	1119	7	Q4QIM6	LM12	1491	34	Q4Q2T2
TB4	1106	8	Q57VN6	TB12	Absent		
TB4b	1102	8	Q57VN8	TC12s	1259	34-S	Q4DPV1
TC4	Absent						
LM5	1194	33	Q4Q490	LM13	1157	34	Q4Q2M2
TB5	1100	10	Q389H9	TB13	1080	4	Q581T0
TC5p	1060	37-P	Q4DPA3	TC13s	1143	34-S	Q4DSF6
LM6	1051	17	Q4QED4	LM14	2525	9	Q4QHT5
TB6	Absent			TB14	1569	11	Q382N5
TC6	Absent			TC14p	1356 (p)	41-P	Q4D911
P2D, Na^+^ transport							
LM7	1109	35	E9AF31	LM15	1097	13	Q4QG01
TB7	1041	9	Q38CW7	TB15	1128	11	Q386H6
TC7s/7p	1039	39-S 39-P	Q4DST4, Q4DU38	TC15s/15p	1099	14-S, 14-P	Q4D434, Q4DGD2
P3A, H^+^ transport				P5A, unknown ion specificity			
LM8	974	18	Q4QDN8	LM16	1244	7	Q4QII2
TB8	920	10	Q388Z2	TB16	1261	8	Q57YG5
TC8s	259 (p)	40-S	Q4CRY5	TC16p	1246	19-P	Q4DK98
TC8p	256 (p)	40-P	Q4DL30				

*Notes.* LM, *L. major* (strain Friedlin); TB, *Trypanosoma brucei brucei* (TREU927, strain 927/4 GUTat10.1); TC, *Trypanosoma cruzi* (strain CL Brener); *T*. *cruzi* chromosome designation “S”, *Esmeraldo like*; *T*. *cruzi* chromosome designation “P”, *Non-Esmeraldo-like*; (p), partial open reading frame. Accession numbers are from the UniProtKB (http://www.uniprot.org) and TriTrypDB (http://tritrypdb.org/tritrypdb/) databases.

P-type ATPases previously characterized are listed in Table 2 and matched with the corresponding protein homologue derived from subsequent genome sequencing data described herein. Information on their function, cellular localization, and transcript expression are also given, if available. Tables 3 and 4 list the P-type ATPases present in eight *Leishmania* and four *Trypanosoma* strains, respectively. All of the prior P-type ATPases reported were detected in the trypanosomatid genomes, indicating the reliability of the current search of the trypanosomatid genomes. The trypanosomatid proteins are organized in Tables 1–4 on the basis of substrate specificity and P-type ATPase subfamily designation. The trypanosomatid parasites possessed ion pumps in the P_1B_ (metal pumps), P_2A_ (SERCA), P_2B_ (PMCA), P_2D_ (Na^+^ pumps), P_3A_ (H^+^ pumps), P_4_ (aminophospholipid translocators), and P_5B_ (no assigned specificity) subfamilies. These represent the P-type ATPase transport functions necessary for survival in the Trypanosomatidae as P-type ATPases for each of these seven subfamilies are found in all *Leishmania* and *Trypanosoma* species included in this analysis. Although H^+^/K^+^-ATPase activity has been described in *L. donovani* and Na^+^/K^+^-ATPase activity reported for *L. mexicana*, *L. amazonensis*, *T. brucei*, and *T. cruzi*, no P_2C_-ATPases which might account for these findings were found in the parasite genomes [25, 35, 49, 72, 95, 111]. No representatives of P_1A_-ATPases, K^+^ transporters or P_3B_-ATPases, Mg^++^ transporters, both found exclusively in prokaryotes, were identified.

**Table 2:** Prior characterization of P-type transport ATPases in the Trypanosomatidae.

Protein	Size (aa)	Prior Homologues [Citation]	Size (aa)	Percent identity	Accession	Localization (stage)	Expression (stage)	mRNA
P2A, Ca^++^ transport (SERCA)								
LD2	1023	LDSERCA [154]	1023	94	Q5S054			
LMx2	1013	Lmaa1 [87, 137]	1031	87	O09489	ER (P, A)	P, A ↑	3.5 kb
TB2	1011	TBA1 [114, 133]	1011	99	P35315	ER (Pc, B)	Pc, B	4.3 kb
TC2s	1006	TcSCA1 [55]	1006	97	O96608	ER (E, T, A)	E, T, A	5.0 kb
P2B, Ca^++^ transport (PMCA)								
TB3	1080	TbPMC2 (TbA2) [90]	1080	100	Q8I919	PM (Pc)	Pc, B ↑	4.43 kb
TB4	1106	TbPMC1 (TbA1) [90]	1106	99	Q8I920	AC (Pc)	Pc, B ↑	4.12 kb
TB4b	1102	TBCA2 [154]	340 (p)	96	Q9XZJ8			
TC3s/3p	1101/1103	Tca1 [88]	1100	90	O60948	AC, PM (E, T, A)	E, T ↑, A ↑↑	4.3 kb
		TCCA2 [154]	341 (p)	98	Q86QH6			
P2D, Na^+^ transport								
LD7	1109	LDCA1 [154]	1047	92	O61136		P, A	>7.5 kb
TB7	1041	TBCA1 [154]	340 (p)	100	Q9XZJ9	PM (Pc, B)	Pc, B ↑	5.0 kb
TC7s/7p	1039	TcENA [66]	1039	100	Q76DT8	PM (E, T, A)	E ↑, T ↑, A	4.5 kb
		TCNA1 [154]	340 (p)	97	Q86QH8			
P3A, H^+^ transport								
LD8	974	LDH1A [98, 99]	974	97	P11718		P, A ↓	5.20 kb
LD9	974	LDH1B [98, 99]	974	95	P12522		P, A	5.75 kb
TB8	920	TbHA3 [91]	920	100	Q6WZI6	PM (Pc, B)	Pc ↑, B	3.58 kb
		TbHA2 [91]	905	92	Q7Z1X2	PM (Pc, B)	Pc ↑, B	3.59 kb
		TBH1 [154]	257 (p)	100	Q9XZJ7			
TB9	912	TbHA1 [91]	912	100	Q86DE0	PM (Pc, B)	Pc ↑, B	3.56 kb
TC10s	898	TCH1 [101]	599 (p)	61	O00931			
		TCH3 [101]	925	64	O15637		E ↑, T	3.8 kb
		TcHA1 [89, 170]	875	65	Q8T7V7	PM, R (E, T, A)	E ↑, T, A	3.86 kb
		TcHA2 [89, 170]	917	64	Q8T7V6	R (E, T, A)	E, T ↑, A	5.35 kb
P4, aminophospholipid translocation								
LD13	1157	LDAPLT1 [154]	1156	89	O61137			
LD15	1097	LdMT [123]	1097	93	Q6VXY9	PM (P)		
TC15s/15p	1099	TCAPLT2 [154]	387 (p)	99	Q86QH7			

*Notes.* aa, amino acids; kb, kilobase pairs; PMCA, plasma membrane calcium ATPase; SERCA, sarcoplasmic-endoplasmic reticulum calcium ATPase; (p), partial sequence; AC, acidocalcisome membrane; ER, endoplasmic reticulum membrane; PM, plasma membrane; R, reservosome; A, amastigote stage; B, bloodstream stage trypomastigote; E, epimastigote stage trypomastigote; Pc, procyclic stage trypomastigote; P, promastigote stage; ↑, upregulated; ↓, downregulated. Accession numbers are from the UniProtKB database (http://www.uniprot.org).

**Table 3:** P-type transport ATPases in genomes of eight species of *Leishmania.*

Protein	Size (aa)	Chromosome	Accession	Protein	Size (aa)	Chromosome	Accession
P1B, Cu^++^, Co^++^ transport				P3A, H^+^ transport			
LM1	1163	33	Q4Q3X8	LM8	974	18	Q4QDN8
LT1	1163	33	LtrL590_330029000	LT8	absent		
LI1	1163	33	A4I980	LI8	974	18	A4HY23
LD1	1163	33	E9BQF1	LD8	49 (p), 84 (p)	18	E9BDY0 + E9BDY1
LA1	1163	33	LAEL147_000688800	LA8	950	18	LAEL147_000273500
LMx1	1246	32	E9B447	LMx8	533 (p)	18	E9ART5LI4
LP1	1224	33	LpaL13_330029400	LP8	927	18	LpaL13_180018000
LB1	1136	33	A4HLS1	LB8	927	18	A4H9Q5
P2A, Ca^++^ transport (SERCA)							
LM2	1023	4	Q95Z93	LM9	974	18	Q4QDN7
LT2	1023	4	LtrL590_040005000	LT9	764 (p)	18	LtrL590_180020500
LI2	1023	4	A4HRZ6	LI9	974	18	A4HY22
LD2	1023	4	E9B7W7	LD9	974	18	E9BDY2
LA2	1023	4	LAEL147_000046100	LA9	158 (p)	18	LAEL147_000273600
LMx2	1013	4	E9AJY3	LMx9	974	18	E9ART6
LP2	1025	4	LpaL13_040005600	LP9	Absent		
LB2	1025	4	A4H3S2	LB9	463 (p), 338 (p)	18	A4H9S1, A4H9S2
P2B, Ca^++^ transport (PMCA)				P4, aminophospholipid translocation			
LM3	1104	7	Q4QIM8	LM11	1279	30	Q4Q767
LT3	872 (p)	7	LtrL590_070012400	LT11	1447	30	LtrL590_300028400
LI3	929	7	A4HTF0	LI11	1279	30	A4I5Q4
LD3	937	7	E9B963	LD11	1279	30	E9BLX0
LA3	875	7	LAEL147_000094200	LA11	1447	30	LAEL147_000570400
LMx3	1104	7	E9AL76	LMx11	1279	29	E9B0Z9
LP3	686 (p)	7	LpaL13_070012000	LP11	1369	30	LpaL13_300026500
LB3	1126	7	A4H514	LB11	1244	30	A4HIF8
LM4	1119	7	Q4QIM6	LM12	1491	34	Q4Q2T2
LT4	Absent			LT12	1491	34	LtrL590_340031100
LI4	294 (p), 863 (p)	7	E9AG74, A4HT82	LI12	1491	34	A4IA39
LD4	328 (p)	7	E9B965	LD12	1491	34	E9BR44
LA4	293 (p), 275 (p)	7	LAEL147_000094300	LA12	1491	34	LAEL147_000729000
LMx4	1119	7	E9AL78	LMx12	1483	33	E9B554
LP4	181 (p), 592 (p)	7	LpaL13_070012300	LP12	1510	20	LpaL13_200028900
LB4	592 (p)	7	A4H516	LB12	1510	20	A4HAY0
LM5	1194	33	Q4Q490	LM13	1157	34	Q4Q2M2
LT5	1224	33	LtrL590_330016200	LT13	1158	34	LtrL590_340037100
LI5	1197	33	A4I8W5	LI13	1157	34	A4IA89
LD5	1197	33	E9BQ35	LD13	1157	34	E9BR98
LA5	1221	33	LAEL147_000675700	LA13	1158	34	LAEL147_000735700
LMx5	1198	32	E9B3T4	LMx13	1158	33	E9B5B1
LP5	1221	33	LpaL13_330015600	LP13	336 (p), 373 (p)	20	LpaL13_200034700
LB5	1194	33	A4HLF4	LB13	666 (p)	20	LbrM.20.2800
LM6	1051	17	Q4QED4	LM14	2525	9	Q4QHT5
LT6	1134	17	LtrL590_170010400	LT14	2510	9	LtrL590_090014400
LI6	1134	17	A4HXD4	LI14	2528	9	A4HU10
LD6	1134	17	E9BD87	LD14	2528	9	E9B9Y1
LA6	1134	17	LAEL147_000247600	LA14	2511	9	LAEL147_000122200
LMx6	1135	17	E9AR29	LMx14	2526	9	E9AMU0
LP6	1113	17	LpaL13_170011200	LP14	2441	NA	LpaL13_000026300
LB6	1113	17	A4H903	LB14	2441	9	A4H5N8
				LM15	1097	13	Q4QG01
P2D, Na^+^ transport							
LM7	1109	35	E9AF31	LT15	1097	13	LtrL590_130020500
LT7	1224	35	LtrL590_350026000	LI15	1097	13	A4HVT2
LI7	1109	35	A4IBA6	LD15	1097	13	E9BBN1
LD7	1109	35	E9BS80	LA15	1097	13	LAEL147_000187900
LA7	1223	35	LAEL147_000770700	LMx15	1104	13	E9APH7
LMx7	1225	34	E9B686	LP15	1096	13	LpaL13_130018700
LP7	1216	34	LpaL13_340025100	LB15	1097	13	A4H7E2
LB7	1104	34	A4HMM8	LB17	1068	13	A4H7E4
P3A, H^+^ transport				P5A, unknown ion specificity			
LM10	952	4	O97198	LM16	1244	7	Q4QII2
LT10	957	4	LtrL590_040016000	LT16	1244	7	LtrL590_070018400
LI10	957	4	A4H5A9	LI16	1244	7	A4HTD0
LD10	957	4	E9B877	LD16	1244	7	E9B9A4
LA10	957	4	LAEL147_000057500	LA16	1244	7	LAEL147_000099500
LMx10	910	4	E9AK92	LMx16	1244	7	E9ALC3
LP10	544 (p), 327 (p)	4	LpaL13_0400179(8)00	LP16	1243	7	LpaL13_070016500
LB10	930 (PG)	4	LbrM.04.1120	LB16	1243	7	A4H553

*Notes.* LA, *Leishmania aethiopica* (strain L147); LB, *L. braziliensis* (strain MHOM/BR/75/M2904); LD, *L. donovani* (strain BPK282A1); LI, *L. infantum* (strain JPCM5); LM, *L. major* (strain Friedlin); LMx, *L. mexicana* (strain MHOM/GT/2001/U1103); LP*, L. panamensis* (strain MHOM/COL/81/L130; LT, *L. tropica* (strain L590); NA, not assigned; (p), partial open reading frame(s); PG, pseudogene (internal stop codons). Accession numbers are from the UniProtKB (http://www.uniprot.org) or from the TriTrypDB databases (http://tritrypdb.org).

**Table 4:** P-type transport ATPases in genomes of four species of *Trypanosoma*.

Protein	Size (aa)	Chromosome	Accession	Protein	Size (aa)	Chromosome	Accession
P1B, Cu^++^, Co^++^ transport				P3A, H^+^ transport			
TB1	961	11	Q387C6	TB8	920	10	Q388Z2
TBL1	961	11	Tb427tmp.47.0023	TBL8	920	10	Tb427.10.12510
TBG1	961	11	D0A5R6	TBG8	Absent		
TC1s/1p	954	39-S, 39-P	Q4DIX9, Q4D866	TC8s	259 (p)	40-S	Q4CRY5
				TC8p	256 (p)	40-P	Q4DL30
P2A, Ca^++^ transport (SERCA)							
TB2	1011	5	Q57Z55	TB9	912	10	Q388Z3
TBL2	1011	5	Tb427.05.3400	TBL9	912	10	Tb427.10.12500
TBG2	1011	5	C9ZPL1	TBG9	912	10	D0A564
TC2s	1006	21-S	Q4DIM7	TC9s	219 (p)	40-S	Q4CP01
				TC9p	484 (p)	40-P	Q4CNY1
P2B, Ca^++^ transport (PMCA)							
TB3	1080	8	D6XMU9	TB10	Absent		
TBL3	1080	8	Tb427.08.1200	TBL10	Absent		
TBG3	1077	8	C9ZUN6	TBG10	Absent		
TC3s/3p	1101, 1103	19-S, 19-P	Q4E3S6, Q4DSJ2	TC10s	898	8-S	Q4DE37
TB4	1106	8	Q57VN6	P4, aminophospholipid translocation			
TBL4	1106	8	Tb427.08.1180	TB11	1196	6	Q584Z5
TBG4	Absent			TBL11	1196	6	Tb427.06.3550
TC4	Absent			TBG11	1196	6	C9ZR23
				TC11s/11p	1217	32-S, 32-P	Q4DXI9, Q4DT76
TB4b	1102	8	Q57VN8				
TBL4b	1102	8	Tb427.08.1160	TB12	Absent		
TBG4b	Absent			TBL12	Absent		
TC4b	Absent			TBG12	Absent		
				TC12s	1259	34-S	Q4DPV1
TB5	1100	10	Q389H9				
TBL5	1100	10	Tb427.10.11620	TB13	1080	4	Q581T0
TBG5	1100	10	D0A4V8	TBL13	1080	4	Tb427.04.1510
TC5p	1060	37-P	Q4DPA3	TBG13	1080	4	C9ZM06
				TC13s	1143	34-S	Q4DSF6
TB6	Absent						
TBL6	Absent			TB14	1569	11	Q382N5
TBG6	Absent			TBL14	1569	11	Tb427tmp.52.0018
TC6p	Absent			TBG14	1569	11	D0A9H6
				TC14p	1356 (p)	41-P	Q4D911
P2D, Na^+^ transport							
TB7	1041	9	Q38CW7	TB15	1128	11	Q386H6
TBL7	1041	9	Tb427tmp.244.2570	TBL15	1128	11	Tb427tmp.02.0850
TBG7	1041	9	C9ZZN4	TBG15	1128	11	D0A6F9
TC7s/7p	1039	39-3, 39-p	Q4DST4, Q4DU38	TC15s/15p	1099	14-S, 14-P	Q4D434, Q4DGD2
				P5A, unknown ion specificity			
				TB16	1261	8	Q57YG5
				TBL16	1261	8	Tb427.08.650
				TBG16	1261	8	C9ZUI4
				TC16p	1246	19-P	Q4DK98

*Notes*. TB, *Trypanosoma brucei brucei* (TREU927 strain 927/4 GUTat10.1); TBL, *Trypanosoma brucei brucei* (strain Lister 427); TBG *Trypanosoma brucei gambiense* (DAL972, strain MHOM/CI/86/DAL972); TC, *Trypanosoma cruzi* (strain CL Brener); *T*. *cruzi* chromosome designation “S”, *Esmeraldo like*; *T*. *cruzi* chromosome designation “P”, *Non-Esmeraldo-like*; (p), partial open reading frame. Accession numbers are from the UniProtKB (http://www.uniprot.org/) and TriTrypDB (http://tritrypdb.org/tritrypdb/) databases.

Although tandemly linked repeated genes are common in the Trypanosomatidae, most of the P-type ATPases are present as single copy genes (Tables 1, 3, 4). A single gene is present in the P_1B_ (metal pumps), P_2A_ (SERCA), P_2D_ (Na^+^ pumps) and P_5B_ (no assigned specificity) subfamilies. Several of the P_2B_ (PMCA) ATPase genes are also single copy and each of the five different genes found in the P_4_ aminophospholipid subfamily are also present in a single copy with the exception of a duplication of the LB15 (LB15–LB17) gene in *L*. *braziliensis*. The only additional P-type ATPases present in linked, repeated assemblies in these parasite genomes are P_2B_ ATPases (PMCA) of *Trypanosoma brucei* (TB3–TB4–TB4b), *Leishmania major* (LM3–LM4) and *L*. *mexicana* (LMx3–LMx4) each separated by a single gene, and P_3A_ ATPases (H^+^ pumps) of *L*. *major* (LM8–LM9), *L*. *infantum* (LI8–LI9) and *T*. *brucei* (TB8–TB9). Other *Leishmania* and *Trypanosoma* species have only a single gene in these two loci, although in many cases gene fragments and pseudogenes are present, indicating the ancestral existence of duplicate genes in these loci. As shown in subsequent sections, differences in the presence and extent of tandem arrays in these P-type ATPase subfamilies as well the presence of loci containing additional P_2B_ ATPase (PMCA) and P_3A_ (H^+^ pumps) genes in *Leishmania* species results in a variable content of P-type ATPases among the different Trypanosomatidae species.

## P-type ATPases in *Leishmania*

Table 3 lists the P-type ATPases present in eight species of *Leishmania* including those causing visceral disease (*L. infantum*, *L*. *donovani*), Old World cutaneous sores (*L*. *aethiopica*, *L. major*, *L*. *tropica*), New World cutaneous lesions (*L. mexicana*), and New World mucocutaneous leishmaniasis (*L. braziliensis*, *L*. *panamensis*). The *L*. *panamensis* and *L*. *braziliensis* sequences belong to the *Viannia* subgenus of *Leishmania*. Alignments of the *Leishmania* ATPases within each subfamily show them to be highly homologous, generally >90% identical in their core sequences, which exclude amino and carboxyl sequences outside the first and last membrane spanning domains. P-type ATPases from Old World species *L. major* and *L. infantum* were usually most closely related in each subfamily and *L. panamensis* and *L. braziliensis* proteins were the least homologous as compared to the other species. The differences in protein sizes within each ATPase family were principally due to alterations in the length of the amino and carboxyl ends of the protein, shorter or longer than the consensus length although in several cases internal sequence was missing. The *Leishmania* ATPases were highly syntenic with identical chromosomal locations, except differences attributable to breakage/fusion rearrangements in chromosomes 8/29 and 20/36 in *L. mexicana* and chromosomes 20/34 in *L. braziliensis* and *L. panamensis* [22, 121]. The ancestral complement of P-type ATPase genes in *Leishmania* appears to be 16. However, rearrangements, deletions, and duplications have altered this complement as only *L. major* has 16 functional P-type ATPase genes. The other *Leishmania* species have reduced numbers of P_2B_ (PMCA), P_3A_ (proton pumps) or P_4_ (aminophospholipid translocators), with only fragments of the original gene remaining, and *Leishmania braziliensis* has an additional gene copy (LB17) in the P_4_-ATPase family, arising from an apparent duplication of LB15.

## P-type ATPases in *Trypanosoma*

Table 4 lists the P-type ATPases present in four *Trypanosoma* species; *Trypanosoma brucei* TREU927, *T. brucei* Lister strain 427, *T. brucei gambiense* DAL972, and *T. cruzi* CL Brener. There are 14 P-type ATPases in *T. brucei* TREU927 and Lister strain 427, 11 in *T. brucei gambiense*, and 12 in *T. cruzi*. *Trypanosoma brucei* TREU927 and Lister strain have repeated genes in the P_2B_ ATPase (PMCA) and P_3A_ (H^+^ pumps) subfamilies, TB/TBL3–TB/TBL4–TB/TBL4b and TB/TBL8–TB/TBL9 respectively, as compared to *T*. *b*. *gambiense* and *T*. *cruzi* which have a single gene in these loci. The three *Trypanosoma brucei* strains also lack a homologue for TC12, one of the five P_4_ (aminophospholipid translocators) present in *T*. *cruzi*. Homologues for TC12 are present in all eight *Leishmania* species (Table 3) and it is an interesting speculation that this protein may be involved in the adaptation to an intracellular environment for the *Leishmania* species and *T*. *cruzi* as the *T*. *brucei* strains have only extracellular forms in their life cycle. There is considerably less heterogeneity in the *Trypanosoma* P-type ATPases in each subfamily, both in protein size and identity, as compared to the differences among the different *Leishmania* species.

The trypanosomatid P-type ATPases are discussed in the following sections on the basis of their subfamily designation, with an emphasis on correlating the genetic and biochemical information available for each P-type ATPase subfamily with the prior reports on their function and transcription provided in Table 2. Data for gene transcription in different life stages are also available for *L. donovani*, *L*. *infantum*, *L*. *major*, *L*. *mexicana*, *T. cruzi*, and *T*. *brucei* and will also be discussed where appropriate [52, 70, 75, 79, 103, 136, 151, 185]. Transcript expression differences between life stages of less than two-fold are considered as constitutive expression, although it should be noted that regulation of P-type ATPase activity in different stages can be regulated on many levels, including transcript stability, protein stability and turnover, post translational modifications, the necessity and activity of obligatory co-factors, and the presence of regulatory domains within many of these P-type ATPases.

## P_1B_-ATPases (3.A.3.5 and 3.A.3.6)

All organisms require trace amounts of metals ions, principally as cofactors in biological catalysis; over 40% of enzymes classified by EC (Enzyme Commission) number, whose three-dimensional structures have been deposited in the Protein Bank Database, are metal dependent [2]. However, in excess, heavy metals are toxic to cells through the binding and inactivation of DNA, lipids, and proteins via the generation of free radicals and reactive oxygen species. Members of the P_1B_-ATPases are critical components to the maintenance of metal homeostasis and catalyze the transport of metal ions such as copper, zinc, silver, lead, cadmium, and cobalt out of the cytosol. P_1B_-ATPases are characterized by 6 (TM1-6) to 8 (TMA, TMB, TM1-6) transmembrane segments (TMs) flanking the large cytoplasmic loop that contains the ATP binding and phosphorylation sites, and the presence of metal binding signature sequences in TM4, TM5 and TM6 which determine ion selectivity. P_1B_-ATPases also possess a diverse array of regulatory cytoplasmic N-terminal and C-terminal metal binding domains that control the enzyme turnover rate, but do not affect metal ion binding to the transmembrane transport sites. Seven P_1B_-ATPase subgroups have been identified and the metal selectivity identified for the first four; P_1B-1_: Cu^+^/Ag^+^; P_1B-2_: Zn^2+^/Pb^2+^/Cd^2+^, P_1B-3_: Cu^2+^; P_1B-4_: Co^2+^, with substrate specificity as yet to be definitively defined for P_1B-5_, P_1B-6_, and P_1B-7_ [3, 4, 152]. Members of these subgroups are characterized by conserved amino acid signature motifs in the transmembrane segments that establish their ion selectivity, and in the number of their transmembrane segments. The P_1B-1_-ATPases (3.A.3.5) are the most widely represented, present in archaea, prokaryotes and eukaryotes. The P_1B-2_, P_1B-3_, and P_1B-4_ subgroups (3.A.3.6) which transport divalent metal ions are restricted to archaea, prokaryotes and plants where they are responsible for metal biotolerance and also function in the hyper-accumulation of divalent metals in some plant species.

A single P_1B_-ATPase is present in each of the *Leishmania* and *Trypanosoma* species described in this report. Those in the *Leishmania* species are 175–292 amino acids longer than their *Trypanosoma* counterparts. This size differential is primarily related to longer amino terminal sequences proximal to the first transmembrane segment present in the *Leishmania* P_1B_-ATPases. However, excluding the cytoplasmic amino and carboxyl terminal portions of the enzymes the *Leishmania* and *Trypanosoma* P_1B_-ATPases are highly homologous, sharing 72–83% identity in this region. The trypanosomatid proteins each exhibit a structural organization characteristic of the Cu^+^ transporting P_1B-1_ subgroup of P_1B_-ATPases; a long N-terminal domain, eight predicted transmembrane domains (TMA, TMB, TM1-6) with the large cytoplasmic loop located between TM4 and TM5, and a relatively short C-terminal domain. The trypanosomatid P_1B_-ATPases also contain the transmembrane signature motifs present in the Cu^+^ transporting P_1B-1_ subgroup; CPCALGLATP in TM4, NX_6_YNX_4_P in TM5, and PX_6_MX_2_SSX_5_N in TM6 [3] Transmembrane signature motifs characteristic of P_1B_-ATPase subgroups with other metal ion specificities were absent in the trypanosomatid proteins. P_1B-1_-ATPases also have an N-terminal consensus regulatory metal binding domain, GMXCXXC, which is present in 2–3 copies in the *Leishmania* species and as a single copy in the *Trypanosoma* species. The trypanosomatid P_1B-1_-ATPases do not possess the Cys or His-rich or (HX)_n_ regulatory metal binding domains associated with other P_1B_-ATPase subgroups but the eight *Leishmania* species each do have an N-terminal CC dipeptide motif that has been associated with metal binding in other subgroups. A phylogenetic comparison with 52 additional P_1B_-ATPases from organisms ranging from archaea and prokaryotes thru vertebrates shows that the trypanosomatid ATPases segregate with the P_1B-1_ Cu^+^ pumps from fungi, plants, vertebrates and invertebrates, and are most closely related to the yeast (*Saccharomyces*, *Trametes*, and *Cryptococcus*) and plant (*Sorghum*) representatives (data not shown).

In archaea and prokaryotes, P_1B_-ATPases function primarily as efflux pumps, conferring resistance to high levels of heavy metals in the environment. Non-photosynthetic eukaryotes appear to lack P_1B_-ATPases specific for divalent metal ions but their P_1B-1_-ATPases have a dual function; they can localize to the plasma membrane and function in copper efflux, conferring metal resistance, or be localized to organellar membranes and supply copper to intracellular compartments to meet metabolic requirements. The *Saccharomyces* copper transporting P_1B-1_-ATPase, CCC2, is located in the membrane of the post-Golgi network where it transfers copper from the copper chaperone Atx1p into the lumen of the Golgi network for insertion into secreted copper-dependent enzymes [62, 184]. In humans, two P_1B-1_-ATPases, ATP7A and ATP7B, localize to the trans-Golgi network and undergo copper-dependent trafficking, but can also be relocated to the plasma membrane or to vesicles proximal to the plasma membrane in various tissues [92]. Defects in ATP7A and ATP7B have been linked to Menke’s syndrome, a copper deficiency disorder resulting from reduced transport of dietary copper, and Wilson’s disease, a disorder characterized by progressive intracellular copper accumulation.

In the absence of biochemical characterization and subcellular localization data, the precise role of the trypanosomatid P_1B-1_-ATPases in their life cycle remains speculative. However, a role similar to CCC2 or ATP7A/B in the transport of copper into the trans-Golgi network or other intracellular organelles for incorporation into metalloproteins seems likely. For instance, a Cu/Zn-type superoxide dismutase is present in *Leishmania* glycosomes [39]. In addition, while the tightly regulated nature of copper metabolism in the human and insect hosts of trypanosomatids would seem to minimize the need for active copper efflux in the plasma membrane, the ability of *Leishmania* species and *T. cruzi* to survive within phagolysosomal vacuoles of macrophages highlight the need for the trypanosomatid P_1B-1_-ATPases to participate in active copper efflux. Macrophages introduce Cu^+^ ions into the lumen of phagolysosomal vacuoles, via the ATP7A copper pump, as part of the toxic antimicrobial milieu present in this compartment [53, 65, 174]. This copper can catalyze the production of reactive hydroxyl radicals capable of damaging microbial lipids, proteins, and nucleic acids. Copper, via adventitious binding to amino acids, can also damage microbes by excluding native metal cofactors from their ligands, particularly in iron-sulfur cluster proteins. Resistance to copper via P_1B-1_-ATPases is required for virulence and survival from macrophage-mediated bacterial clearance in *Escherichia coli*, *Pseudomonas*, pneumococcus, and *Salmonella* infections [74, 78, 147, 174]. Transcripts of the *Trypanosoma cruzi* P_1B-1_-ATPases are upregulated over three-fold in the intracellular amastigote stage present in host cells versus the extracellular epimastigote in the insect vector, emphasizing its importance in an intracellular environment for *T. cruzi* [103]. However, in *Trypanosoma brucei*, which lacks an intracellular stage in the human host, there is little difference in P_1B-1_-ATPase transcript and protein levels between insect and human stages [75]. Although there is little difference in transcript levels between intracellular amastigote forms and insect promastigote stages for *L. mexicana* and *L. major*, the presence of multiple regulatory copper-binding domains in the amino terminal segments of the *Leishmania* P_1B-1_-ATPases may provide an alternative mechanism for increasing efflux activity under the high copper conditions encountered within macrophage phagolysosomes [52, 79].

## P_2A_-ATPases (3.A.3.2)

The maintenance of intracellular calcium homeostasis is critical to living cells. Calcium is an integral component in signaling pathways that control many aspects of cellular function, including transcription, motility, cell division, apoptosis, metabolism, and signal transduction. Free calcium level within cells is maintained at micro-molar concentrations more than three orders of magnitude lower than intracellular calcium storage sources or the extracellular environment. Vertebrate cells utilize two sources of calcium for signaling, the sarcoplasmic reticulum and the extracellular space but intracellular parasites such as *Leishmania* and *T*. *cruzi* must contend with the low free calcium content of host cells. As a consequence, the trypanosomatid parasites have an additional calcium storage compartment, an acidic, calcium and phosphorous rich organelle known as an acidocalcisome [41]. Two classes of P-type Ca^2+^-ATPases participate in regulation of intracellular calcium levels; the P_2A_-ATPases, also known as the sarcoplasmic-endoplasmic reticulum calcium ATPases (SERCA), and P_2B_-ATPases known as plasma membrane calcium ATPases (PMCA), though PMCA pumps can also be found in intracellular organelles such as Golgi vesicles, vacuoles, and acidocalcisomes. SERCA pumps are found within intracellular organelles, notably the sarcoplasmic and endoplasmic reticulum, where they mediate Ca^2+^ sequestration, maintaining low intracellular Ca^2+^, and providing a recruitable Ca^2+^ store for intracellular signaling. The P_2A_-ATPases in most organisms have a high affinity for calcium and are inhibited by sub-micromolar concentrations of the sesquiterpene lactone thapsigargin. In trypanosomatids, calcium similarly functions as a second messenger to control intracellular signaling but also mediates several events unique to these organisms [42, 129]. *Trypanosoma brucei* maintains a low intracellular concentration of free Ca^2+^ and increases in calcium concentration are implicated in release of variant surface glycoprotein (VSG) from the plasma membrane [20, 141]. The periodic and synchronized release of VSGs is critical to evasion of antibody-mediated immune responses during infection. Host cell invasion by *T*. *cruzi* trypomastigotes and *L*. *amazonensis* is also dependent on increased cytosolic calcium levels [87, 107, 182].

The trypanosomatid parasites have multiple Ca^2+^-ATPases, including a single representative of the P_2A_-SERCA pumps and 2–4 members of the P_2B_-PMCA pumps. The SERCA pumps in the three trypanosomatid genomes initially sequenced (LM2, TB2, TC2s; Table 1) share 65–76% identity and 76–85% homology in pairwise comparison. The additional seven *Leishmania* genomes sequenced each include a single copy SERCA gene (Table 3) and their relatedness with LM2 ranges from 86–97% identity and 91–98% homology. Similarly, the genomes of additional *Trypanosoma* species (Table 4) also include a single P_2A_-ATPase and comparisons of these sequences shows 76–99% identity and 85–99% homology. SERCA pumps with a high degree of identity (87–99%, Table 2) to TC2s, TB2, and LM2 have been characterized in *T. cruzi*, *T. brucei*, *L. donovani*, and *L. mexicana amazonensis*. The *T. brucei* SERCA, TBA1 (Table 2), has high affinity for calcium, is sensitive to nano-molar concentrations of thapsigargin, is expressed in intracellular microsomal fractions but not in plasma membrane fractions, and significantly increases microsomal calcium activity when over-expressed but plasma membrane Ca^2+^-ATPase activity is unchanged [114, 133]. As expected for SERCA function, as a housekeeping protein, TBA1 is constitutively expressed in both procyclic and bloodstream trypomastigotes. The *T*. *cruzi* SERCA, TcSCA, contains sequence motifs unique to SERCA pumps, localizes to the endoplasmic reticulum in amastigotes, epimastigotes, and trypomastigotes, and is constitutively expressed in all three developmental forms. TcSCA expression in yeast rescues mutants deficient in the Golgi and vacuolar Ca^2+^-ATPase, PMC1, and TcSCA restores growth of PMC1 mutants on Mn^2+^ containing media, suggesting a role in Mn^2+^ uptake [55]. The *L*. *mexicana amazonensis* SERCA pump, Lmaa1, is located in the endoplasmic reticulum of both amastigotes and promastigotes, its expression is upregulated 2–4 fold in amastigote stages, and overexpression of Lmaa1 increases infectivity both *in vitro* and *in vivo* [87, 137]. In contrast to TBA1, the SERCA pumps from *T. cruzi* and *L. amazonensis* are reported to be relatively insensitive to thapsigargin; their sequences exhibit poor homology to the amino acids in transmembrane region 3 implicated in thapsigargin binding. *Trypanosoma cruzi* membrane ATPase activity in the presence of calcium was inhibited 24.9 ± 1.5% by the addition of 1 μM artemisinin to the assay and by 25.8 ± 3.9% by 1 μM thapsigargin [104]. Although the *T. cruzi* SERCA pump is thapsigargin insensitive when expressed in yeast, there are three additional calcium ATPases present in the *T. cruzi* genome and thapsigargin-sensitive calcium stores have been documented in *T. cruzi* [30, 40, 55]. The presence of thapsigargin sensitive calcium stores in other trypanosomes has also been reported [102].

## P_2B_-ATPases (3.A.3.2)

Plasma membrane Ca^2+^-ATPases (PMCA) are high affinity calcium pumps that participate in the maintenance of low intracellular Ca^2+^ levels characteristic of eukaryotic cells by exporting Ca^2+^ from the cytosol to the extracellular environment. Vertebrates possess multiple PMCA genes and each transcript can be alternatively spliced to produce multiple variants that are developmentally regulated in a tissue- and cell-specific manner [180]. In more primitive eukaryotes (fungi, protozoa), members of the PMCA family also localize to intracellular organelles such as Golgi and vacuolar membranes. PMCAs of animals and plants are autoinhibitory proteins that are activated by calmodulin binding to a cytoplasmic C-terminal domain in animals and an N-terminal domain in plants [19, 142]. No consensus calmodulin-binding domain (CaMBD) exists but CaMBDs are typically 15–30 amino acids that have a net positive charge, possess moderate hydrophilicity and hydrophobic anchor residues, and have a propensity to form amphipathic α-helices [110].

Phylogenetic analysis identifies three distinct groups of PMCAs in trypanosomatids, with each group located on a different chromosome; (i) LM3-4, TB3-4-4b, Tc3s/3p, (ii) LM5, TB5, Tc5p, and (iii) LM6, each group presumably with different physiological roles. The first group exists as tandemly linked copies in *Leishmania*, two copies on chromosome 7, although only partial sequences or gene fragments are noted for some *Leishmania* species at this locus, and in *T*. *brucei*, three copies on chromosome 8 (Tables 3 and 4). *Trypanosoma brucei gambiense* and *T*. *cruzi* only have a single Ca^2+^-ATPase representative in this first group. The second group has only a single gene in each of the trypanosomatid parasites, and the third group is only present in *Leishmania*. PMCA pumps with a high degree of identity (90–100%, Table 2) to TB3, TB4, TB4b, and TC3s/3p have been characterized in *Trypanosoma brucei* and *T. cruzi*. *Trypanosoma brucei* TbPMC1 (TB4) and TbPMC2 (TB3) can complement yeast mutants whose vacuolar calcium ATPase and Ca^2+^/H^+^ antiporter are deleted, and suppress their calcium hypersensitivity [90]. TbPMC1 localizes to acidocalcisomes where it is responsible for calcium sequestration in this organelle and TbPMC2 localizes to the plasma membrane. Both proteins are upregulated in bloodstream trypomastigotes but plasma membrane calcium ATPase activity is equivalent in both forms [90, 114]. A partial sequence (TBCA2) homologous to TB4b has been reported but not characterized [154]. *Trypanosoma cruzi* Tca1 (TC3s/3p, Table 2) co-localizes with the vacuolar H^+^-ATPase to the plasma membrane and to intracellular vacuoles whose properties are consistent with acidocalcisomes. Northern analysis shows that Tca1 is >6-fold more abundant in amastigotes and >3-fold more abundant in trypomastigotes than in epimastigotes [88]. Although calmodulin-binding domains could not be identified in Tca1 (TC3s/3p) or TbPMC2 (TB3), calmodulin stimulation of plasma membrane Ca^2+^-ATPase activity has been demonstrated in *T*. *cruzi*, *T*. *brucei*, and *L*. *mexicana* [12–14]. In *Leishmania*, a high-affinity plasma membrane Ca^2+^-ATPase activity, responsible for calcium extrusion from *L*. *donovani* promastigotes, has been reported but not functionally characterized [96]. No genes of the second (LM5, TB5, Tc5p) or third groups (LM6) of trypanosomatid PMCA pumps have been characterized to date.

## P_2D_-ATPases (3.A.3.9)

The physiologic dependence of living cells on K^+^ ion participation in many metabolic activities, as well as the toxic effects of a high intracellular Na^+^ content, requires active transport mechanisms to regulate K^+^ and Na^+^ ion levels and maintain high K^+^ and low Na^+^ within cells. In lower eukaryotes, fungi, and bryophytes, a combination of inward K^+^ ion channels, K^+^ uptake proteins, and P-type ATPase efflux and import pumps capable of transporting either Na^+^ or K^+^ has evolved to serve this function [138]. These ATPases, classified as type P_2D_-ATPases, function to confer tolerance to high concentrations of Na^+^ and K^+^ in the environment by exchanging Na^+^ (or K^+^) for H^+^. This efflux can be either across the plasma membrane or into intracellular organelles to sequester sodium. In fungi and lower eukaryotes, this will also necessitate efflux of the imported H^+^ ions via a proton pump (see P_3A_-ATPases). In invertebrates and vertebrates Na^+^ and K^+^ homeostasis is regulated by a counter-transporting P-type Na^+^/K^+^-ATPase which couples the export of three Na^+^ ions with the import of two K^+^ ions. The Na^+^/K^+^-ATPase thus requires the presence of both Na^+^ and K^+^ for activity [109]. The Na^+^/K^+^-ATPase, classified as a type P_2C_-ATPase, is inhibited by ouabain and insensitive to furosemide, whereas the yeast P_2D_ Na^+^ pumps are typically insensitive to ouabain and sensitive to furosemide. In animal cells, the electrogenic nature of Na^+^/K^+^-ATPase activity also produces a large transmembrane electrochemical gradient of sodium ions that is harnessed to drive the secondary transport of nutrients and other substrates [109].

During their life cycle, the trypanosomatid parasites are exposed to differing challenges in regulating Na^+^ and K^+^ ion homeostasis. In the human host, high serum Na^+^ (135–150 mM) and low serum K^+^ concentrations (3.6–5.6 mM) are encountered during growth in blood and low Na^+^ (5–15 mM) and high K^+^ (140–155 mM) concentrations are present in the intracellular environments seen by *T. cruzi* and *Leishmania* amastigotes. While information on the ionic environment in tsetse flies and sandflies, vectors for *T. brucei* and *Leishmania*, is lacking, it is reasonable to expect that Na^+^ and K^+^ concentrations in their insect hosts during the digestion of the parasite acquired blood meal will be similar to those seen in human serum, as has been shown in the gut of reduviid bug vectors of *T. cruzi* after feeding [76]. However, the identity of the protein(s) responsible for the active transport of Na^+^ and K^+^ ions in the trypanosomatids during the life cycle has been controversial; both P_2C_-ATPases with properties similar to vertebrate Na^+^/K^+^-ATPases and P_2D_ ATPases similar to fungal and plant Na^+^ ATPases have been reported [25, 26, 35, 36, 49, 66, 95, 154, 165]. The difficulty in distinguishing these two types of pumps has been a reliance on using sensitivity to ouabain, a widely used Na^+^/K^+^-ATPase inhibitor, as distinguishing criteria, resulting in the discrepancies in interpretation between different investigators. Ouabain does not have absolute specificity for the Na^+^/K^+^-ATPase and can inhibit additional cellular functions as well as other P-type ATPases, albeit to a lesser extent and at higher concentrations. Cystamine transport is inhibited by 60% in *Saccharomyces* by 200 μM ouabain although *S. cerevisiae* lacks an Na^+^/K^+^-ATPase [131]. Ouabain in micromolar concentrations also significantly inhibits the human non-gastric H^+^/K^+^-ATPase [157]. Ecto-ATPase activity in *Streptococcus sanguis* and divalent cation stimulated ATPase activity in *Staphylococcus aureus* are also slightly inhibited by micromolar concentrations of ouabain [77, 93]. Therefore, genetic analysis of the trypanosomatid P-type ATPase sequences and characterization of their biochemical properties are more reliable criteria for classifying these pumps.

A single sodium pump is present in each of the trypanosomatid genomes (LM7, TB7, TC7) and these are highly homologous: pairwise alignments of the *T*. *brucei*, *T*. *cruzi* and *Leishmania* protein sequences show 65–73% identity and 77–82% homology within the region between the first and last trans-membrane segments, excluding amino and carboxyl terminal amino acids. Homologues for each of these genes have also been cloned and partially characterized (Table 2). Examination of their amino acid sequences clearly indicates that the trypanosomatid sodium pumps are related to the P_2D_-ATPase fungal sodium efflux pumps and not the Na^+^/K^+^-ATPases present in animal cells, the P_2C_-ATPases. A phylogenetic analysis has previously identified these trypanosomatid ATPases as members of the P_2D_-ATPases and an updated analysis performed for this work confirms this report [154]. Although the trypanosomatid genome sequence annotation in the TriTrypDB database identifies these as putative calcium motive ATPases, they clearly segregate with the P_2D_-ATPases in the current analysis. The trypanosomatid ATPases are most closely related to the fungal Na^+^ and K^+^ efflux pumps and not to the fungal high affinity K^+^ or Na^+^ uptake proteins recently described [16]. None of the amino acids which confer ouabain specificity to Na^+^/K^+^-ATPases are conserved in the trypanosomatid P_2D_ sodium pumps, a finding which questions the specificity of ouabain inhibition of ATPase activity in the trypanosomatid parasites [126]. A DSYGQ motif located in the TM7–TM8 extracellular loop of Na^+^/K^+^- and H^+^/K^+^-ATPases, which is essential for their interaction with the accessory β-subunit, ion translocation and ATP hydrolysis, also cannot be found in the trypanosomatid sodium pumps [11, 29]. In addition, no homologues of this accessory β-subunit, required for P_2C_ Na^+^/K^+^-ATPase activity, have been identified in the trypanosomatid genomes. The amino acids responsible for Na^+^ and K^+^ binding within the transmembrane segments (TM4, TM5, TM6, TM8, and TM9) that form the cation pore of Na^+^/K^+^-ATPases are conserved in each of the trypanosomatid sodium pumps [116]. However, these residues are conserved in fungal P_2D_-ATPases as well, and simply reflect a common evolutionary origin of the ability of P_2C_- and P_2D_-ATPases to transport Na^+^ and K^+^. There is strong similarity in the trypanosomatid P_2D_-ATPase sequences (matching at 6–9 amino acids) to a conserved motif, MIEALHRRKK, located in ATPase conserved region g, that is present in fungal P_2D_-ATPase ENA pumps but absent in Na^+^/K^+^ P_2C_-ATPases [138]. Two residues shown to be essential for full activity in the *Zygosaccharomyces rouxii* ENA1 ATPase, D852 in TM7 and E981 in TM10, are also conserved in the trypanosomatid P_2D_-ATPases, but are not present in Ca^2+^-, Na^+^/K^+^-, or H^+^-ATPases [172].

Biochemical properties of the trypanosomatid Na^+^- and K^+^-ATPase activities are also most consistent with a classification as P_2D_-ATPases. In yeast, the primary function of their P_2D_-ATPases is sodium or potassium efflux in conditions of high concentrations of these ions. A similar role is likely for the trypanosomatid sodium pumps. ATPase activity in *Trypanosoma cruzi* epimastigote membranes is stimulated by addition of Na^+^ ions and does not require the presence K^+^ for activity, as do Na^+^/K^+^-ATPases, and this stimulation is ouabain-insensitive and furosemide-sensitive [26]. The characterization of a TC7p/TC7s homologue in the *T. cruzi* Tulahuen strain, TcENA (Table 2), provides biochemical validation that the trypanosomatid P_2D_-ATPases function as Na^+^ or K^+^ efflux pumps similar to plant and fungal sodium pumps [66]. The ATPase activity of TcENA expressed in mammalian cells was stimulated by either Na^+^ or K^+^ and is insensitive to ouabain. *Trypanosoma cruzi* epimastigotes over-expressing TcENA also showed increased tolerance to Na^+^ stress. TcENA is localized to the plasma membrane and is expressed in all parasite stages, although transcript levels were lowest in the intracellular amastigote stages which are exposed to the lowest sodium concentrations encountered in the life cycle. A partial gene sequence for TB7 has been reported (TBCA1) and it also localizes to the plasma membrane and is upregulated in trypomastigote stages [154]. *Leishmania amazonensis* ATPase activity in promastigote plasma membranes is also stimulated by Na^+^ ions, does not require the presence K^+^ for activity, and is ouabain-insensitive and furosemide-sensitive [36]. Heme, a hemoglobin degradation product prominent in insect guts during blood meal digestion, where high Na^+^ would also be encountered, modulates this (Na^+^ + K^+^) ATPase by increasing intracellular H_2_O_2_ production which activates a protein kinase C signaling pathway to increase (Na^+^ + K^+^) ATPase activity [35, 37, 135]. In addition to its role in Na^+^ efflux through the plasma membrane the trypanosomatid P_2D_-ATPases may also function in cyclic AMP-protein kinase A (PKA) signaling and other intracellular signaling pathways. The PKA regulatory subunit immunoprecipitates with multiple P-type ATPases (TcENA, Tc7s, Tc16p) in trypomastigote membranes and P-type ATPases may play a role in anchoring PKA to the plasma membrane [10]. In humans, the Na^+^/K^+^-ATPases also serve as an anchor for a signalosome, containing specific binding motifs for proteins such as caveolin, phosphoinositide 3′ kinase, ankyrin, and AP2 adaptor complex. When bound to ouabain, the Na^+^/K^+^-ATPases signalosome can activate Src family tyrosine kinases and transduce signals via multiple pathways independent of its pumping function [83, 181]. A consensus caveolin binding motif, FxxxxFxxF, is present in the amino terminus proximal to TM1 in the trypanosomatid Na^+^ pumps.

## P_3A_-ATPases (3.A.3.3)

Plasma membrane H^+^-ATPases are found in plants, fungi, protozoa and archaebacteria. They are a major determinate in the maintenance of cytosolic pH and by pumping H^+^ ions out of the cell they generate and maintain an electrochemical H^+^ gradient across the plasma membrane (PMF, proton motive force), which is used to energize secondary transport of solutes [108]. In animal cells, this electrochemical gradient is based on Na^+^ ions and is controlled by P_2C_ Na^+^/K^+^-ATPases. P_3A_-ATPases are characterized by the presence of an auto-inhibitory domain which regulates protein activity. In fungi, this domain is located in the C-terminal cytoplasmic portion of the protein and interacts intra-molecularly to lock the pump in a low activity state. In plants, both the C-terminal and N-terminal domains are involved in controlling H^+^-ATPase activity [44]. Binding of 14–3–3 proteins to plant H^+^-ATPases or glucose to fungal H^+^-ATPases neutralizes this interaction and moves the pump to a high activity state. This regulation is principally dependent on the phosphorylation of serine and threonine residues in the C-terminal auto-inhibitory domain of both fungi and plants [63, 118]. Tandem phosphorylation of an adjacent Serine-Threonine amino acid pair in the C-terminal tail of the yeast Pma1 H^+^-ATPase mediates glucose-dependent activation of Pma1 [80].

Multiple P_3A_-ATPases are present in the trypanosomatid species. *Leishmania* has H^+^-ATPase genes in two chromosomal locations; a multi-copy gene locus on chromosome 18 and a single copy H^+^-ATPase gene on chromosome 4 (Tables 1 and 3). The genome sequences of *L. major* and *L*. *infantum* each contain three proton pumps; a tandemly linked pair on chromosome 18 and the separate, single copy gene on chromosome 4. There are only one (*L*. *tropica*, *L*. *panamensis*, *L*. *braziliensis*) or two (*L*. *donovani*, *L*. *aethiopica*, *L*. *mexicana*) complete H^+^-ATPases gene sequences found in the genomes of the other *Leishmania* species (Table 3). However, there are partial gene sequences or pseudogenes in the analogous genomic locations for each of the H^+^-ATPases missing from the *L*. *major* and *L*. *infantum* complement, indicating that at one time, three P_3A_-ATPase genes were likely present in each *Leishmania* species. The reasons for this loss of H^+^-ATPases in the genome sequences of some *Leishmania* species is not clear, but perhaps multiple copies of these genes are only necessary *in vivo* and that prolonged *in vitro* cultivation has led to their loss. A tandemly linked pair of H^+^-ATPases has been demonstrated in isolates different from those characterized in the genome databases for *L*. *braziliensis*, *L*. *donovani*, *L*. *mexicana*, and *L*. *tropica* [100]. The two *L*. *donovani* Ethiopian L82 strain H^+^-ATPases (Table 2) have been cloned and sequenced [98, 99].

*Trypanosoma brucei brucei* strains TREU927 and Lister 427 each also contain a tandemly linked pair of proton pumps on chromosome 10, but only a single H^+^-ATPase is found at this locus in the *Trypanosoma brucei gambiense* genome (Table 4). Three H^+^-ATPases have been identified at this locus on chromosome 10 in a strain Lister 427 29–13 cell line as well (Table 2) [91]. The separate, single copy H^+^-ATPase locus on a different chromosome as seen for *Leishmania* species is absent in the *T*. *brucei brucei* strains and in *Trypanosoma brucei gambiense*. The *T*. *cruzi* strain CL Brener genome only contains partial H^+^-ATPase sequences for two genes on chromosome 40 and a complete H^+^-ATPase gene on chromosome 8 (Tables 1 and 4). However, two linked H^+^-ATPase genes have been cloned and characterized from *T*. *cruzi* strain Y (Table 2) and four tandemly linked H^+^-ATPase genes have been reported for *T*. *cruzi* strain Sylvio-X10/7 [89, 101]. The identity of these trypanosomatid ATPase genes as H^+^-ATPases has been confirmed by functional complementation of the yeast proton pump by LDH1A and LDH1B from *L*. *donovani*, TcHA1 and TcHA2 from *T*. *cruzi*, and TbHA1, TbHA2, and TbHA3 from *T*. *brucei* [61, 89, 91].

The function of the trypanosomatid H^+^-ATPases is analogous to the roles identified for yeast and plant proton pumps. Maintenance of cytosolic pH and proton motive force driven transport of nutrients has been demonstrated for both amastigote and promastigote stages of *L. donovani* [58–60, 85, 112, 186–188]. The internal pH and membrane potential are comparable for the two *Leishmania* morphologic forms despite the differing pH environments they are exposed to; pH 4.5–5.0 for amastigotes within the phagolysosomal vacuoles of macrophages and pH 7.0–7.5 for promastigotes in the midgut of sandfly vectors and in culture. Amino acid uptake in *L. major* is also dependent on proton pump generated membrane potential and intracellular pH in *L. mexicana amazonensis* is regulated by plasma membrane H^+^-ATPase activity [97, 169]. *Trypanosoma brucei* proton pump activity regulates membrane potential (deltapsi) in both procyclic and bloodstream forms of the parasite as shown by plasma membrane depolarization by H^+^-ATPase inhibitors [166]. A *T*. *brucei* H^+^-ATPase also regulates intracellular pH in procyclic trypomastigotes and bloodstream trypomastigotes [164]. Three P-type H^+^-ATPases (TbHA1-3, Table 2), cloned and sequenced from *T*. *brucei*, localize to the plasma membrane of procyclic forms and the plasma membrane and flagellum of bloodstream forms, complement a yeast strain deficient in endogenous H^+^-ATPase activity, are upregulated in procyclic forms, and RNA interference of their expression resulted in growth inhibition. Knockdown of TbHA1 and TbHA3 lowered internal pH (pH_*i*_) and slowed recovery of pH_*i*_ after acidification [91]. The regulation of cytoplasmic pH and plasma membrane potential in *T*. *cruzi* epimastigotes, trypomastigotes and amastigotes is similarly dependent on plasma membrane H^+^-ATPase activity and participation of a P-type H^+^-ATPase in the acidification of internal organelles has been shown [148, 162, 163, 165]. A tandemly linked pair of H^+^-ATPases, TcHA1 and TcHA2 (Table 2), has been characterized in *T*. *cruzi* [89]. The two proton pumps were expressed in all *T*. *cruzi* forms although TcHA1 is most abundant in epimastigotes and TcHA2 is expressed predominantly in trypomastigotes. TcHA1 and an N-terminal truncated TcHA2 complemented yeast deficient in H^+^-ATPase activity and were localized to the yeast plasma membrane. In all *T*. *cruzi* developmental forms, both TcHA1 and TcHA2 are located in intracellular compartments, principally reservosomes, and TcHA1 is additionally present in the plasma membrane [170].

The *Leishmania*, H^+^-ATPases are regulated at their C-terminus like their yeast counterparts. *Leishmania donovani* proton pumps LDH1A and LDH1B (Table 2) are differentially regulated; LDH1A is constitutively expressed while LDH1B is significantly upregulated in intracellular amastigote forms. The two proteins are highly homologous, differing at only 20 amino acids with 15 of those differences occurring in the COOH-terminal 37 amino acids [98, 99]. This carboxyl terminal tail region contains multiple potential serine and threonine phosphorylation sites including the proposed *Leishmania*-specific phosphorylation motifs FS and NxS [117]. Functional complementation of a *Saccharomyces cerevisiae* strain deficient in endogenous H^+^-ATPase activity, support of yeast growth at low pH, and ability to acidify media demonstrate that LDH1A and LDH1B encode proton pumps. LDH1A and LDH1B encode a COOH-terminal auto-inhibitory domain as COOH-truncated peptides support increased rates of growth in yeast, enhanced media acidification, increased enzyme activity *V*_max_ and decreased *K*_m_. This regulatory domain mediates differing function properties; LDH1A, but not LDH1B, supports yeast growth at pH 3 and LDH1A shows a greater ability to acidify media. Deletion of the last eight amino acids from LDH1B permits growth at pH 3 and increases media acidification, swapping of the COOH-tails between LDH1A and LDH1B results in LDH1A (with LDH1B tail) unable to support yeast growth at pH 3 and LDH1B (with LDH1A tail) now able to support growth at pH 3. Replacement of the COOH-terminal eight amino acids of LDH1B with those from LDH1A also confers the ability to support growth at pH 3 [61]. Potential phosphorylation motifs are present in the COOH-terminal regions of other *Leishmania* H^+^-ATPases. Multiple Ser and Thr residues are present in all of the proton pumps for the additional *Leishmania* species described here, including a Ser-Thr pair in each, as well as NxS and FS motifs in most of the *Leishmania* species. The COOH-terminal tails of the *Trypanosoma* proton pumps also contain multiple Ser and Thr residues and NxS/FS motifs but no Ser-Thr pairs. However, the *T*. *cruzi* proton pump, TcHA1, is apparently not regulated via a COOH-terminal auto-inhibitory domain as COOH-terminal truncation of TcHA1 did not affect its ability to complement yeast deficient in H^+^-ATPase activity [89].

## P_4_-ATPases (3.A.3.8)

The aminophospholipid translocases are a large and diverse group of P-type ATPases that appear to be ubiquitous in eukaryotes where the expression of multiple P_4_-ATPases is common. A unique feature of eukaryotes is the asymmetrical nature of their membranes and aminophospholipid translocases are responsible for maintaining the asymmetrical distribution of lipids in the plasma membrane, trans-Golgi network and endosomal system membranes by their ability to “flip” phospholipids such as phosphatidylserine (PS), phosphatidylethanolamine (PE), and phosphatidylcholine (PC) between bilayer leaflets to increase PS and PE exposure on the cytosolic leaflet and PC and sphingolipid exposure on the extracellular leaflet [34, 82, 134]. The P_4_-ATPases work in synergy with other ATP-dependent and ATP-independent protein families, such as ATP binding cassette (ABC) transporters and flippases respectively, to effect this non-random phospholipid distribution in eukaryotic membranes. This asymmetric lipid arrangement is critical to cellular function as it confers different biophysical properties and physiological roles to the two sides of the bilayer. P_4_-ATPases are also essential to membrane biogenesis by generating lipid asymmetry in the new membrane as it transverses from its origin in the endoplasmic reticulum through the Golgi network. In addition to their role in vesicular trafficking, P_4_-ATPases have also been implicated in cell signaling events, apoptosis, blood clotting, phagocytosis, cell polarity and growth, adhesion, migration, lysophospholipid transport, and apical barrier function [1, 120].

Different members of the P_4_-ATPases can differentiate phospholipids based on their head groups and lipid backbones. Yeast possesses five P_4_-ATPases; Drs2p transports PS with a weaker affinity for PE, Dnf1p, Dnf2p, and Dnf3p transport PC and PE, and the transport specificity of Neo1p appears to be PE with a weaker activity towards PS [120, 158]. Yeast P_4_-ATPases also exhibit specificity in their intracellular location; yeast Dnf1p and Dnf2p localize to the plasma membrane and early endosomes, Dnf3p and Drs2p are located in the trans-Golgi network, and Neo1p is present in the trans-Golgi network and late endosomes [120]. The P_4_-ATPases require at least three accessory (β subunit) molecules, which form heterodimeric complexes with the P_4_-ATPases in membranes and are required for their stability, proper intracellular targeting, and transport activity and specificity. In yeast, Cdc50p complexes with Drs2p and Neo1p, Lem3p/Ros3p complexes with Dnf1p and Dnf2p, and Crf1p binds Dnf3p. Five classes of P_4_-ATPase ATPases (14 total), analogous to those in yeast, with different substrate specificities, a similar diverse array of cellular membrane localizations, and accompanied by accessory chaperone proteins, (CDC50A, CDC50B, and CDC50C) are also present in humans [1].

A total of 14 P_4_-ATPases are present in the trypanosomatid genomes initially sequenced (Table 1); five in *L. major* and *T. cruzi*, and four in *T. brucei*. Alignment of their amino acid sequences identifies five groups; (i) LM11, TC11s/11p, and TB11; (ii) LM12 and TC12s; (iii) LM13, TB13 and TC13s; (iv) LM14, TB14, and TC14p; and (v) LM15, TB15, and TC15s/15p. The first two groups show higher homology to each other than to other groups, a finding also true for the last two groups. This result is confirmed by phylogenetic analysis with 42 additional P_4_-ATPases (not shown) wherein the trypanosomatid sequences from the first two groups (LM11-TB11-TC11s/11p and LM12-TB12) segregate on a separate branch of the phylogram, as do the last two groups (LM14-TB14-TC14p and LM15-TB15-TC15s/15p), while LM13, TB13 and TC13s segregate together on a branch that includes yeast Neo1p. Homologous proteins in each of these five P4-ATPase groups are present in all the other *Leishmania* and *Trypanosoma* species analyzed as well, with the exception of a homologue for LM12 and TC12 which is missing in all of the *Trypanosoma brucei* strains (Tables 3 and 4). In view of the close correlation in the P-type ATPase complements of the trypanosomatid parasites, the lack of a *T. brucei* homologue of LM12 and TC12s is somewhat perplexing. It may be hypothesized that this absence is due to the lack of an intracellular amastigote stage in the *T. brucei* life cycle. Perhaps LM12 and TC12s mediate membrane alterations necessary for entry into their resident cells or unique to survival in an intracellular environment. The close relationship between yeast neo1p and LM13, TB13, and TC13s also implies a similar cellular role and intracellular location, in endosomes, as for Neo1p, for this group of trypanosomatid P_4_-ATPases. The need for P_4_-ATPases in the plasma membrane and within the trans-Golgi network suggests that the other trypanosomatid P_4_-ATPases function there.

Except for the P_4_-ATPase group represented by LM15, physiological roles and membrane locations for the trypanosomatid P_4_-ATPases have not been elucidated. Although residues involved in determining phospholipid specificity have been identified in yeast, plant and human phospholipid translocators there is insufficient homology between these proteins and the trypanosomatid P_4_-ATPases to make any definitive conclusions as to their substrate specificities [56, 71, 140]. An *L. donovani* homologue of LM15, LdMT (93% identity, 96% similarity), localizes to the plasma membrane and mediates uptake of glycerophospholipids and the lysophospholipid analogue miltefosine (hexadecylphosphocholine), a highly effective oral anti-leishmanial drug [122, 123]. Resistance of *L. donovani* promastigotes and amastigotes to miltefosine is characterized by mutations to LdMT, which decrease internalization of miltefosine and other phospholipid analogues [123, 149]. Resistant parasites also exhibit changes in membrane lipid composition and alterations in fatty acid, lipid, and sterol metabolism [5, 127, 128, 150]. The efficacy of miltefosine against *T. cruzi* and to a lesser extent versus *T. brucei*, suggest a similar functional role for their LM15 homologues, TC15s/15p (54% identity, 69% similarity to LM15) and TB 15 (48% identity, 61% similarity to LM15) [31, 146].

The characterization of a functional homologue, LdRos, of the Lem3/CDC50 family in *L. donovani* (strain MHOM/ET/67/HU3) reveals a similar requirement for accessory β subunits in trypanosomatid P_4_-ATPase function [124]. LdRos is located in the plasma membrane with LdMT and its presence is necessary for proper LdMT trafficking to the membrane, LdMT translocation activity, and uptake of miltefosine (conferring resistance if absent or mutated). Three members of the Lem3/Ros family of P4-ATPase accessory molecules, homologous to those present in yeast and humans, are also present in each of the *Leishmania* and *Trypanosoma* genomes described in this report. The LdRos protein homologue in *L. major* genome is 93% identical to LdRos and homologous proteins in *T. brucei* and *T. cruzi* are 53% and 55% identical to LdRos.

P_4_-ATPases participate in entry and intracellular survival of *Leishmania*. Phagocytosis of *Leishmania* amastigotes and infective stationary phase promastigotes is enhanced by increased exposure of phosphatidylserine on the outer membrane [167]. The presence of increased PS is normally a signal of cellular apoptosis, inducing phagocytosis by polymorphonuclear neutrophils, granulocytes, and macrophages, and acting to suppress immune activation and inflammatory responses by these cells. Intracellular pathogens such as *Leishmania* exploit this process by mimicking the apoptotic programmed cell death phenotype to gain entry into target cells and evade immune responses [9, 171]. Although the protein that mediates this increased PS exposure in *Leishmania* has not been identified, the dependence of PS exposure on an ATP driven process in *Leishmania* has been demonstrated, an observation consistent with inhibition or reduction of membrane P_4_-ATPase PS translocating activity [161].

## P_5A_-ATPases (3.A.3.10)

The P_5_-ATPases are the least well understood subfamily of the P-type ATPases, which is surprising as they appear to be ubiquitous among eukaryotes. P_5_-ATPases appear to be specific for eukaryotes as no P_5_-ATPases have been identified in bacterial or archaeal species to date. P_5_-ATPases group into two subfamilies, P_5A_ and P_5B_, based on the composition of a characteristic signature sequence present in their fourth transmembrane domain, which corresponds to the PEGLP sequence of P_2A_-ATPases that participates in forming a Ca^2+^ binding site [160]. In P_5A_-ATPases this motif contains the sequence PP(D/E)LPxE and in P_5B_-ATPases the two negative charged residues have been replaced with hydrophobic residues, PP(A/V)LPAx, with x denoting a hydrophobic residue [105]. The loss of two negative residues in a critical transmembrane segment implicated in ion specificity will impact ion binding properties and suggests that P_5A_- and P_5B_-ATPases may differ in their ion specificities. The P_5A_-ATPases are present in all eukaryotic lineages but P_5B_-ATPases are not found in three eukaryotic lineages; excavates, which includes *Leishmania* and *Trypanosoma*, entamoebas, and land plants.

Multiple studies associate P_5A_-ATPases to the endoplasmic reticulum (ER), early Golgi network, vacuoles, and secretory vesicles [33, 48, 69, 156, 168, 175]. Ion specificity has not been definitively ascribed for P_5_-ATPases and loss of function mutants in both yeast and plants exhibit pleiotropic effects with diverse phenotypes [153]. Available evidence implicates P_5_-ATPases in protein folding, maturation, degradation, and secretion through maintenance of ER homeostasis. The *Saccharomyces* Cod1 mutant is deficient in the regulation of hydroxymethylglutaryl-coenzyme A degradation, an integral ER protein, and disruption of this P_5A_-ATPase (also known as Spf1p) yields a glycosylation defective phenotype, perturbs cellular calcium homeostasis, and increases expression of Kar2p, an ER stress chaperone, and calcineurin-activated genes, indicating secretory calcium store depletion [32, 33, 155, 156]. Spf1p/Cod1p expression is regulated by the unfolded protein response and Spf1p/Cod1p mutants activate the unfolded protein response and are defective in the ER-associated degradation of mis-folded proteins and in glycoprotein processing [168]. Microsomes derived from Spf1p deleted yeast exhibit reduced levels of Mn^2+^ and microsomes from Spf1P overexpressing yeast have increased Mn^2+^ as compared to wild type [28]. Deletion mutants of the *Schizosaccharomyces pombe* P_5A_-ATPase, SPAC29A4.19c, are hypersensitive to elevated calcium in the absence of the vacuolar Ca^2+^-ATPase, Pmc1p, a condition that can be suppressed by overexpression of another Ca^2+^-ATPase, Pmr1p. Double deletion mutants of SPAC29A4.19c and Pmr1p are hypersensitive to Mn^2+^ depletion in the culture medium. *Schizosaccharomyces* SPAC29A4.19c deletion mutants are also hypersensitive to the antiarrhythmic drug amiodarone, which disrupts Ca^2+^ homeostasis [54]. Mutants of the P_5A_-ATPase of *Arabidopsis thaliana*, MIA, exhibit abnormal morphology, altered cell wall structure, imbalances in cation homeostasis, and changes in the expression of secretory proteins [69].

Mutations in ATP13A2, a human neuronal P_5B_-ATPase, are linked to the lysosomal storage disorder neuronal ceroid lipofuscinosis and neurodegenerative diseases such as Kufor-Rakeb syndrome, Parkinson’s disease, and hereditary spastic paraplegia, and are characterized by lysosomal and mitochondrial dysfunction [21, 38, 47, 130]. In *Dictyostelium* amoeba, phagocytic bacterial predators found in soil, deletion of kil2, a P_5B_-ATPase present in phagolysosomal membranes, prevents killing and growth on *Klebsiella* bacteria and this defect is rescued by addition of Mg^2+^ ions, suggesting that kil2 is a magnesium pump [81]. However, the dependence of polyamine uptake in *Caenorhabditis elegans* on the presence of the P_5B_-ATPase plasma membrane transporter, CATP-5, demonstrates the existence of an additional potential function for the P_5B_-ATPases [64].

The perturbations in Ca^2+^, Mg^2+^, and Mn^2+^ homeostasis seen in many studies has led to speculation that P_5_-ATPases transport one or more of these ions. An alternative hypothesis is that the P_5_-ATPases function to move lipids or organic molecules across membranes to assist in the generation of membrane vesicles, participate in lipid recycling and/or lipid distribution in membranes, or to move glycolipid precursors, needed for synthesis of *N*-linked oligosaccharides or GPI-anchors, from the cytosol to the lumen of the ER [143–145]. Supporting this hypothesis is the observation that the P_5_-ATPases are most closely related to the P_4_-aminophospholipid translocating ATPases among the P-type ATPases [8]. Unfortunately, definitive evidence to distinguish between these potential substrates for P_5A_- or P_5B_-ATPases is lacking at this time.

P_5_-ATPase genes are present in the all of the Trypanosomatidae examined (Tables 1, 3, 4). Their predicted proteins are of similar size (1244–1261 aa) and highly homologous, sharing 70–81% identity and 80–88% similarity in sequence alignments comparing the *Leishmania* and *Trypanosoma* species. Transcripts of the trypanosomatid P_5_-ATPases do not vary significantly between different life cycle stages, and currently there is no information available on their location or function in the trypanosomatids. The trypanosomatid P_5_-ATPases each contain the PPELPME signature sequence present in the fourth transmembrane segment that is characteristic of all P_5A_-ATPases [105]. A phylogenetic analysis (not shown) of the P_5_-ATPases from the trypanosomatid parasites and 32 additional P_5_-ATPases, 16 P_5A_-ATPases and 16 P_5A_-ATPases, from protozoa, fungi, plants, invertebrates and vertebrates indicates that the trypanosomatid P_5_-ATPases segregate with the P_5A_-ATPases which include MIA, SPAC29A4.19c, and Spf1p/Cod1p. It is reasonable to propose functions for trypanosomatid P_5_-ATPases similar to those described for MIA, SPAC29A4.19c, and Spf1p in the endoplasmic reticulum and/or Golgi network necessary for protein maturation, secretion, and degradation.

## P-type ATPases as drug targets

Chemotherapeutic interventions for *Leishmania* and *Trypanosoma* pathogens are often inadequate due to factors that include the toxic side effects for many of the currently utilized drugs, the cost of recommended treatment regimens, the requirement for long treatment courses, the need for parenteral administration, high therapeutic failure rates, and rising drug resistance in these parasites. The P-type ATPases of *Leishmania* and the trypanosomes represent an ideal class of proteins to exploit in the development of new drugs that can circumvent these limitations. The P-type ATPases described in this report are essential proteins in cellular metabolism, are for the most part constitutively expressed in the different developmental stages of these pathogens, and as surface membrane proteins possess readily accessible extracellular domains. Additionally the H^+^- and Na^+^-ATPases described here differ significantly from their mammalian homologues, the H^+^/K^+^- and Na^+^/K^+^-ATPases. The feasibility of targeting ion pumps is evident from the clinical use of proton pump inhibitors (omeprazole, lansoprazole, etc.) to inhibit the human gastric H^+^/K^+^-ATPase and decrease stomach acidity, cardiotonic steroids (ouabain, digoxin, etc.) for the treatment of congestive heart failure and heart arrhythmia by specific inhibition of the heart Na^+^/K^+^-ATPase, artemisinin compounds for the treatment of malaria via inhibition of the parasite sarcoplasmic/endoplasmic reticulum calcium ATPase (SERCA), and clotrimazole, another SERCA inhibitor, as an anti-mycotic agent [183]. The antiarrhythmic, amiodarone, as well as miltefosine and posaconazole have been shown to exert anti-leishmanial and anti-trypanosomal activity through disruption of calcium homeostasis [15]. There are multiple examples of the efficacy of P-type ATPase inhibitors on *Leishmania* and *Trypanosoma* parasites; artemisinins have anti-trypanosomal activity, omeprazole inhibits *L. donovani*, thapsigargin inhibits the SERCA pump of *T. brucei*, the Na^+^-ATPase of *L*. *amazonensis* is sensitive to the diuretic furosemide (Lasix), pentamidine inhibits the plasma membrane Ca^2+^-ATPase of *T*. *brucei*, the aminophospholipid translocator, LdMT, in *L*. *donovani* is required for miltefosine transport and miltefosine inhibits the Na^+^-ATPase of *T*. *cruzi* [6, 13, 73, 104, 114, 125, 146]. Moving forward, the exploitation of P-type ATPase inhibitors as new therapeutic options in treating *Leishmania* and trypanosome infections should be a priority.

## Conclusion

The identification of the genes responsible for ATP-dependent ion movements should facilitate future studies of their structure and function. Importantly, the exploitation of P-type ATPase genes as drug targets should be advanced as their presence offers the possibility of exploiting differences in their ion pumps for the biologically based design of new therapeutic interventions for these infections.

## Conflict of interest

The author declares that he has no conflict of interest.
